# Heterogeneity of hepatocellular carcinoma: from mechanisms to clinical implications

**DOI:** 10.1038/s41417-024-00764-w

**Published:** 2024-03-18

**Authors:** Fatema Safri, Romario Nguyen, Shadi Zerehpooshnesfchi, Jacob George, Liang Qiao

**Affiliations:** Storr Liver Centre, The Westmead Institute for Medical Research, The University of Sydney, Westmead, NSW 2145 Australia

**Keywords:** Cancer models, Liver cancer

## Abstract

Hepatocellular Carcinoma (HCC) is one of the most common types of primary liver cancer. Current treatment options have limited efficacy against this malignancy, primarily owing to difficulties in early detection and the inherent resistance to existing drugs. Tumor heterogeneity is a pivotal factor contributing significantly to treatment resistance and recurrent manifestations of HCC. Intratumoral heterogeneity is an important aspect of the spectrum of complex tumor heterogeneity and contributes to late diagnosis and treatment failure. Therefore, it is crucial to thoroughly understand the molecular mechanisms of how tumor heterogeneity develops. This review aims to summarize the possible molecular dimensions of tumor heterogeneity with an emphasis on intratumoral heterogeneity, evaluate its profound impact on the diagnosis and therapeutic strategies for HCC, and explore the suitability of appropriate pre-clinical models that can be used to best study tumor heterogeneity; thus, opening new avenues for cancer treatment.

## Introduction

Based on GLOBOCAN’s 2020 report, liver cancer accounted for 830180 deaths globally and accounts for 4.7% of the total cancer cases worldwide [1]. Of all the total cancer cases, liver cancer is the 6th most common cancer with an age-standardized rate (ASR) of 8.6 whereas it is the 3rd most common cause of cancer-related deaths. Asia constitutes around 73% of the liver cancer cases prevailing in the last 5 years, followed by Western and Northern Africa (9.4%), Europe (8.7%), and Northern America (5.2%) [2]. A retrospective study based on observing the survival years of 32,556 cases confirmed the overall survival rates of less than 8% for both males and females [3]. In Australia, 2424 liver cancer-related deaths were estimated in 2022 with a 5-year survival of just 22% [4]. Furthermore, the incidence and mortality rates are two to three times higher in men as compared to women [5, 6]. The incidence of liver cancer in the USA has more than tripled since 1980. Additionally, the incidence of liver cancer is projected to rise significantly by more than 55% with an estimated 1.3 million deaths globally by 2040 [1, 7]. The rising trend of liver cancer incidence and its associated mortality poses a great healthcare burden and calls for attention.

**Fig. 1:**
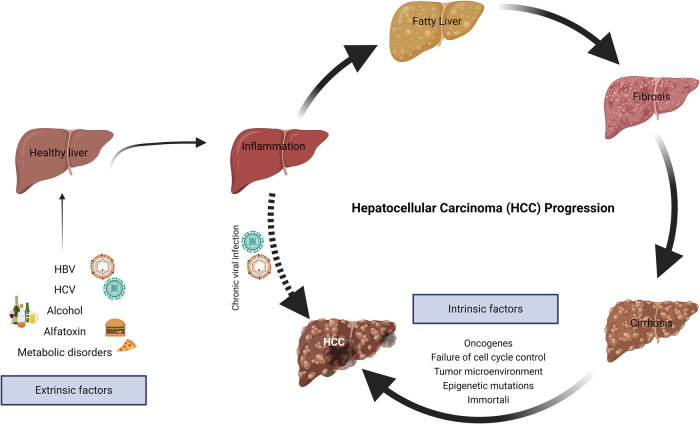
Schematic representation of multi-stage HCC development from common etiological factors.

Primary liver cancer originates within the liver and, depending on the cellular type and pathological morphology, it can be hepatocellular carcinoma (HCC), intrahepatic cholangiocarcinoma (CAA), angiosarcoma, hemangiosarcoma, and hepatoblastoma [8–10]. Whereas, secondary liver cancer refers to those metastasized to the liver from other sites of the body such as colorectal cancer or pancreatic cancer [11]. Among all, hepatocellular carcinoma (HCC) is the most common form of primary liver cancer, contributing to 90% of the total liver cancer cases. It originates from hepatocytes with a doubling time of 4–5 months [12]. HCC predominantly occurs in the background of chronic liver diseases such as hepatitis B and C viral infections, metabolic (dysfunction) -associated fatty liver diseases (MAFLD, previously known as non-alcoholic fatty liver disease, NAFLD), and cirrhosis [13, 14]. Other commonly seen risk factors for HCC include aflatoxin exposure, obesity, diabetes mellitus, and alpha 1-antitrypsin deficiency [15]. Over the past years, MAFLD has become an increasingly important cause of HCC [16]. In addition to these causative agents, several intrinsic factors such as genetic mutations, tumor microenvironment, clonal evolution of cancer cells and epigenetic changes also contribute to the development of HCC. Regardless of etiological factors, non-resolving liver inflammation is a key predisposing factor for the development of primary liver cancer, in that approximately 90% of HCC cases are associated with chronic inflammation leading to fibrosis, cirrhosis, and ultimately HCC (Fig. 1).

HCC is notorious for its rapid progression and poor survival rates, which are often attributed to the presence of heterogeneous subpopulations of cancer cells in the tumor. While there are multiple treatments available for HCC, their effectiveness is limited due to the complexity and heterogeneity of the disease. In this review, we will delve into the factors inducing tumoral heterogeneity in HCC and the associated underlying mechanisms, and propose novel pre-clinical models that can be used to best study HCC heterogeneity, with the ultimate goal of exploring novel approaches that can be used to tackle tumor heterogeneity.

## Tumor heterogeneity

**Fig. 2:**
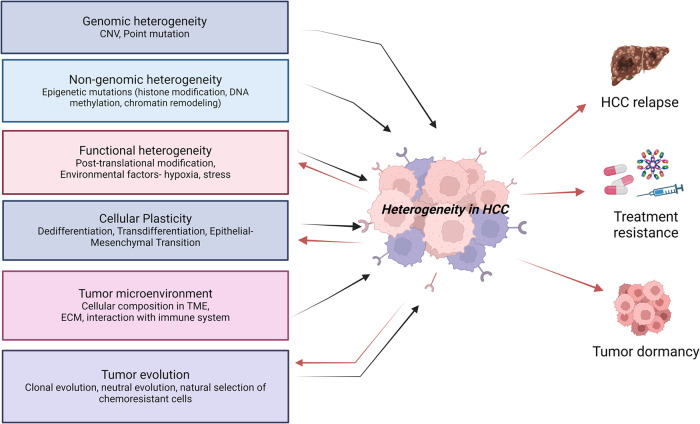
Factors causing cellular heterogeneity in hepatocellular carcinoma and associated clinical implications.

Tumor heterogeneity encompasses the presence of various cell subgroups within a tumor or among tumors sharing the same histopathological subtype. These subpopulations of cells exhibit different genetic and physical characteristics, potentially leading to distinct biological behaviors [17, 18]. At the population level, tumor heterogeneity is observed between tumors of different patients or between different tumor nodules of the same patient (inter-tumoral heterogeneity), within the same tumor nodule (intratumoral heterogeneity) [19], before and after treatments (spatiotemporal heterogeneity) [20] and cancers with different etiologies such that HCCs caused by hepatitis virus infection and alcohol consumption may exhibit distinct cellular and molecular features (etiological heterogeneity) [21]. Heterogeneity is a well-observed phenomenon in HCC resulting in cellular, molecular, functional, and lineage diversity and is connoted to be a result of varying genetic diversity in patients and environmental factors. The mechanisms of heterogeneity are multifactorial including genomic mutations, tumor microenvironment (TME), evolution and reprogramming of cancer cells, the transition from non-cancer to cancer cells, and epigenetics changes, all contributing to the clonal evolution of cancer thereby causing genomic, molecular, and functional heterogeneity in the tumor [22–24]. The multifaceted development of HCC not only contributes to the complexity of the disease but also plays a crucial role in clinical observation such as treatment resistance, tumor dormancy, and recurrence after the initial treatment (Fig. 2).

### Intratumor heterogeneity in HCC

Intratumoral heterogeneity (ITH) reflects the presence of diverse cellular subpopulations, and distinct molecular signatures within the same tumor [17, 23]. Cellular heterogeneity in HCC has been long known based on their histology, cytological findings, morphology, and microscopic growth patterns [24]. These findings have been the basis for developing HCC subtypes and grading the tumor into diverse groups, allowing us to tailor the treatments and predict the prognosis of HCC patients. Despite decades of research and innovation of techniques, a clear understanding of HCC heterogeneity has been a great challenge. In addition to the varying mutations driving a heterogeneous population of cells in HCC, tumor cells exhibit different cell surface markers and have differential expression of genes and dysregulated cellular pathways. For example, a study by Yamashita et al. using 40 HCC patient tissues identified the presence of multiple cell subpopulations within the same tumor, based on the presence of cell surface markers (EpCAM^+^ AFP^+^, EpCAM^+^ AFP^−^, EpCAM^−^ AFP^+^, EpCAM^−^ AFP^−^) [25]. Similarly, a study by Gao et al. involving 55 spatially distinct samples from 10 HCC patients confirmed the presence of 39.7% heterogeneous mutation in all the samples [26]. Recent studies using single-cell RNA sequencing (scRNA-seq) and cell cluster analysis revealed ITH of the global molecular profiles suggesting that the expression of varying biomarkers reflects the different cellular origins in HCC [27]. Furthermore, these biomarkers contribute to the expression of different signaling pathways which means that multi-targeted therapeutic approaches are needed to achieve a cure for heterogeneous cancer.

ITH presents several challenges in the treatment of patients with liver cancer. Firstly, the presence of multiclonality, or the coexistence of multiple distinct tumor cell populations within a single tumor means that the tumor cells may have varied biological behavior, and this compounded with the sampling errors makes accurate diagnosis and pathological analysis difficult. Secondly, the presence of complex and heterogeneous subpopulations in tumor mass implies that these heterogeneous tumor cells may exhibit asynchronous responses to the same treatment, hence leading to treatment resistance and relapse. This is reflected by the lack of targeted therapies that can effectively target the multiple mutated driver genes present within the tumor. The currently available immunotherapy has opened new avenues for treating HCC, however, the monotherapy targeting PD-1 receptor and CTLA-4 receptors showed low anti-tumor response, underscoring a need to combine immunotherapies with other chemotherapies. It is now understood that the complex TME and different genetic and phenotypic characteristics in tumor cells exhibit different biomarkers which are responsible for the varying responses to immunotherapy and difficulty in targeting the entire tumor [28, 29].

### HCC heterogeneity at the molecular level

With the advent of molecular technologies, researchers over the past many years have identified numerous signaling pathways and genetic and epigenetic changes that are aberrantly expressed during the development and progression of HCC (Table 1). Aberrant activation or dysregulation of signal transduction pathways in the liver affects the proliferation, survival, differentiation, and apoptosis of parenchymal liver cells, consequently resulting in tumorigenesis. The most significantly altered pathways include Notch, Wnt/β-catenin, PI3/AKT/mTOR, Ras/Raf/MAPK, JAK/STAT, and ubiquitin-proteasome [30–35]. Consequently, multiple molecular-targeted drugs (e.g., Sorafenib, Regorafenib, etc.) have been developed to inhibit the activity of relevant signaling pathways. However, the current targeted therapies only offer limited efficacy and the tumor often becomes insensitive to the drugs after a few rounds of exposure [36], and the development of drug resistance can be attributed to the presence of heterogeneous subpopulations of the cancer cells and their mechanism of drug efflux [37]. Clearly, there is a need to study the interwoven networking of pathways in HCC thereby finding better molecular markers to target the entire tumor.

**Table 1:** Recent significant findings enhancing the understanding of heterogeneity in HCC.

Studies	Experimental techniques used	Findings/ Remarks
Xue et al. [91]	Exome and whole genome sequencing	Common mutations shared by all HCC lesions varied from 8% to 97%, indicating significant ITH. Confirmed correlation between the tumor size and ITH.
Ho et al. [92]	Single-cell RNA sequencing	A rare subpopulation of CD24^+^/CD44^+^ cells in HCC was identified, demonstrating the association of the CTSE gene in imparting stemness to HCC.
Ding et al. [93]	Next-generation sequencing and methylome analysis	Studied the genomic and epigenomic alterations and confirmed that various signaling pathways (JAK-STAT) and a combination of mutations (in TP53 and 17p) provide with HCC progression and replicative advantages.
Karagonlar et al. [40]	HuH-7 cell lines	KLF-4 induces EpCAM^+^/CD133^+^ LCSCs and modulates de-differentiation
Sun et al. [84]	Single-cell RNA sequencing	CCL5 chemokine is associated with immune evasion (by recruiting Tregs) in HCC and is overexpressed in circulating tumor cells
Yao et al. [94]	Single-cell RNA sequencing	AURKA and EZH2 expression contributes to tumor proliferation, HCC migration, and invasion.
Zhao et al. [95–100]	Spatial transcriptomics	Defined 6 marker genes as the prognostic signature in HCC. Tumors with histological similarities showed significant differences in transcription profiles.

*CD* Cluster of differentiation, *CTSE* Cathepsin E, *JAK-STAT* Janus kinase/signal transducers and activators of transcription, *TP53* Tumor protein p53, *KLF-4* Krüppel-like factor 4, *EpCAM* epithelial cell adhesion molecule, *CCL5* C-C Motif Chemokine Ligand 5, *Tregs* Regulatory T cells, *AURKA* Aurora kinase A, *EZH2* Enhancer of zeste homolog 2.

### Liver cancer stem-like cells in HCC heterogeneity

HCC is a diverse and complex cancer comprising both non-stem cancer cells and cancer stem cells (CSCs). Well-documented evidence has explained HCC development as a result of clonal evolution resulting from the accumulation of mutations over the period [23]. Solid evidence also confirms the role of CSCs in the pathogenesis of HCC [33] making this subpopulation of cells an ideal target for therapy.

According to the stem cell theory of tumor evolution, CSCs exhibit similar properties to normal stem cells including embryonic stem cells in that they are capable of unlimited self-renewal, division, and differentiation that begets a strong survival advantage [34]. Consequently, CSCs increase the chance of recurrence, metastasis, and therapeutic resistance [35].

**Fig. 3:**
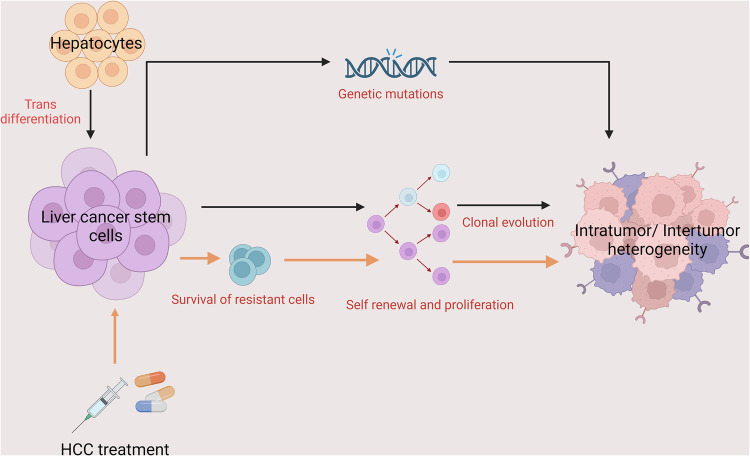
Role of LCSCs in heterogeneity of hepatocellular carcinoma.

Over decades, multiple surface markers for liver cancer stem-like cells (LCSCs) have been identified, such as EpCAM, CD13, CD24, CD44, CD47, CD90, CD133, ICAM1, LGR5, OV6, ALDH, and CK19 [33]. Although LCSCs positive for any of these markers show features for CSCs, no marker is unique for LCSCs. In fact, these molecules are not simply the biomarkers of CSCs but are also functionally linked to multiple biological features of the CSCs and their tumorigenic abilities such as drug resistance, proliferation, migration, metastasis, plasticity, and contribution to tumor heterogeneity. It is known that LCSCs and non-cancer stem cells dynamically switch phenotypes over time, a phenomenon known as “phenotype switching”. This has been demonstrated in animal models using lineage tracing and scRNA-seq analysis [37, 38]. The plasticity of CSCs is of particular relevance to tumor heterogeneity, and this phenomenon is a very unique and common feature of LCSCs. For example, in the studies by Zheng et al. [39] and Karagonlar et al. [40], its was found that non-stem cells (or marker-negative cells) can gain the features of CSCs by trans-differentiation, either spontaneously or by over-expression of certain transcription factors such as KLF-4, and this “trans-differentiation” of CSCs contributes to the development of tumor heterogeneity. Key mechanisms of how LCSCs contribute to the progression and development of heterogeneity in HCC are shown in Fig. 3. With emerging technologies such as scRNA-seq and spatial transcriptomics, our understanding of how LCSCs contribute to the heterogeneity of liver cancer has improved. However, many questions remain, including the cellular origins of HCC and LCSCs, the factors that drive the plasticity of LCSCs, underlying mechanisms, and consequently, how we can tackle tumor heterogeneity via intervening CSC plasticity. Indeed, the complexity of ITH, mostly driven by the existence of varying tumor cell populations each exhibiting unique biological characteristics and responses to treatment, further complicates the matter. Since LCSCs are believed to dictate the cellular clones within the tumor bulk and drive tumor heterogeneity, they are an ideal therapeutic target (Fig. 3). However, there are still many challenges that must be overcome for the clinical adoption of using LCSCs as the target for liver cancer therapy, and these may include the identification of specific targetable molecules, efficient delivery approaches, and accurate assessment of the therapeutic efficiency.

## Heterogeneity impacts the accurate diagnosis of HCC

Despite multiple advanced imaging techniques and blood serum markers have been introduced into clinical practice [41], accurately diagnosing HCC at an early stage has been a significant challenge. Imaging modalities, such as ultrasound, computed tomography, and magnetic resonance imaging, are commonly used tools for detecting liver lesions, but they often lack sensitivity, especially with small lesions or in the context of liver cirrhosis [42]. Meanwhile, false-positive diagnosis may also occur, leading to unnecessary invasive procedures or undue anxiety for patients. Thus, molecular signatures such as serum and tumor biomarkers that can offer heightened sensitivity and specificity for early and accurate diagnosis of HCC are much desirable diagnostic tools.

A well-known diagnostic biomarker for HCC is alpha-fetoprotein (AFP), a crucial glycoprotein in fetal development. The serum level of AFP usually ranges around 0-40 ng/mL in healthy adults but can rise to >400 ng/mL in HCC [43–45]. AFP is so far the most widely used biomarker for HCC diagnosis. However, it is not the most recommended test for early detection of HCC because of its poor specificity (its elevation has also been reported in other conditions such as liver cirrhosis, gastric cancer [46], pancreatic and lung cancer [47]) and poor sensitivity (a proportion of HCC patients do not have AFP secretion [48]). The mosaic pattern of AFP expression in HCC stems from the heterogeneity of HCC tissues. Similarly, other markers such as Des-γ-Carboxy Prothrombin (DCP) (also known as prothrombin induced by vitamin K absence-II, PIVKA-II) cannot accurately diagnose the HCC in patients with vitamin K deficiency, poor nutrition with alcohol abuse, and those using oral anticoagulants [49]. Furthermore, the combination of the commonly used serum markers (e.g., AFP, DCP, and AFP-L3) does not seem to significantly enhance the sensitivity and specificity of early HCC diagnosis [50–53]. In a US-based study by Marrero et al., the combination of AFP, DCP, and AFP-L3 could identify 43% of HCC patients who had AFP levels below 10.9 ng/mL but showed a sensitivity of only 60% [52]. Combination of AFP and DCP showed an improved sensitivity but the specificity decreased significantly [53]. These data indicate tumor heterogeneity as a confounding factor for accurate diagnosis.

The impact of heterogeneity on accurate diagnosis of HCC is also reflected in the complexity of the established tissue markers. For example, each of Glypican-3 (GPC3), glutamine synthetase (GS), and heat shock protein 70 (HSP70) (recommended by the European Association for the Study of Liver to distinguish the dysplastic nodules and well-differentiated HCCs smaller than 2 cm [50]) have shown significant diagnostic value for HCC: GPC3 is positive in ~80% HCCs [54, 55] including in 63.24% of AFP-negative HCCs [55]; has an overall sensitivity of 97.7%, and an overall specificity of 94.7% [56]; GS is positive in 72.4% HCCs [57]; HSP70 is positive in 56.3% of HCCs [58] with a sensitivity of 78.2% and specificity of 100% [59]. However, a combination of GPC3, GS, and HSP70 showed reduced sensitivity for the detection of HCC (25%) [60]. Furthermore, the positivity of these markers shows considerable variations across different tumor stages. For example, GPC3 is positive in 37.3% of Stage I HCC, 71.9% of Stage II HCC, and 92.9% of Stage III HCC [61]. The complexity and inefficiency of the commonly used biomarkers for HCC are also indicated in a recent study by Wang et al. which confirms the presence of diverse ways of tumor evolution and these biomarkers may be just expressed in a partial subpopulation of the HCC while the remaining subpopulation may remain undiagnosed [62]. Hence, accurate diagnosis and subsequent treatments of HCC require a thorough understanding of the inter- and intratumoral heterogeneity. As a result, there is a constant need for identifying and validating the HCC-specific markers that can potentially be diagnostic and screening targets.

## Heterogeneity imparts treatment resistance

Currently, the US Food and Drug Administration (FDA) and European Medicines Agency (EMA) have approved multiple first-line therapies for advanced HCC, including multi-target tyrosine kinase inhibitors (TKIs) such as Sorafenib and Lenvatinib which downregulate signal transduction promoting cell proliferation, angiogenesis, cell migration, and survival [63, 64], and immune checkpoint inhibitors (ICIs) such as atezolizumab, Bevacizumab or Durvalumab/Tremelimumab, which bind to specific ICIs programmed cell death ligand-1 (PDL-1), or cytotoxic T-lymphocyte-associated protein 4 (CTLA-4) to inhibit the downregulation of immune response [65–67]. However, the efficacy of these agents is limited and drug resistance frequently develops, due to multiple mechanisms such as the presence of CSCs, alteration in the signaling pathway, and altered drug efflux mechanism in the HCC [31, 68]. Importantly, cancer heterogeneity contributes to poor treatment outcomes as the heterogeneous cancer cells within a tumor may develop into mixed subclones of cancer cells with different genetic and molecular signatures, eventually responding differently to the same treatments [34, 69], complicating the choice for the most effective treatment for patients. The negative impact of tumor heterogeneity on cancer therapy is supported by a recent study in acute myeloid leukemia where cellular hierarchy composition was closely associated with drug sensitivity of targeted therapies. Clinically, patients with higher tumor diversity scores showed significantly poorer overall and progression-free survivals [70]. Deciphering heterogeneous cell subpopulations and identifying tumor-specific molecular signatures are crucial for advancing personalized cancer therapeutics. To achieve this, there is a pressing need for improved translational research models that can bridge the gap between basic discoveries and clinical applications.

## Current approaches for dissecting HCC heterogeneity

Earlier techniques employed to study tumor heterogeneity include microscopic examination of the tumor tissues, the use of cancer cell lines with varying genetic and pathological backgrounds, immunohistochemistry staining, and bulk RNA sequencing to examine the different populations of cells within the tumor. Coupling the more advanced technologies such as scRNA-seq, spatial transcriptomics, pathway enrichment analysis, and whole genome sequencing, with clinically relevant translational models that can faithfully recapitulate the true pathogenesis of HCC will have great potential in dissecting the interactions of different cell types and their biological behaviors within the tumor bulk.

### Organoids

Organoids are in vitro, self-organizing, stem cell-based 3D tissue models that mimic the native in vivo tissue, allowing researchers to recapitulate the biological, structural, and genetic complexity of an organ [71, 72]. Over the past decades, organoids have been used to study tumor complexities, test drug efficacy, and study the pathogenesis of tumor development [73–76]. This 3D model can be applied to study the cellular heterogeneity in tumors thereby attaining a comprehensive understanding of the interactions among different subpopulations in tumors. The heterogeneous population of LCSCs is one of the major hurdles in the development of an effective therapeutic outcome and leads to drug resistance. Healthy liver organoids have been developed from the liver stem cells wherein various differentiation factors such as dexamethasone, Notch signaling inhibitor, and BMP without Rspo1 [77] are employed to drive the stem cells toward developing into hepatocyte-based organoids. These organoids can be used to study how stemness is imparted to non-stem-like, well-differentiated hepatocytes, thus allowing us to understand the functionalities of stem cells and CSCs in forming heterogeneous tumors as well as how drug resistance occurs in the background of heterogeneous cell populations. The great potential for using organoids in studying tumor heterogeneity is reflected in recent studies where organoids from primary and metastatic colorectal cancer (CRC) were studied by transcriptome and histopathology and the intra- and inter-tumoral heterogeneity of CRC was confirmed [78, 79]. We envisage that organoids derived from HCC patient tissues of various backgrounds will form a promising translational model for deciphering the mechanisms of tumor heterogeneity, particularly the role of LCSCs in HCC.

### Precision-cut liver slice

Precision-cut Liver Slice (PCLS) is an ex vivo model obtained by slicing human liver tissues. The key feature of PCLS is that the multicellular histoarchitecture, the spatial structural relations of the original cell populations, as well as the genetic characteristics of the original organ are well-preserved and maintained for a period of time in vitro, making it a great model for studying heterogeneous subpopulations in HCC. PCLS has been extensively used in studying drug response and toxicity, elucidating the stages of fibrosis and the efficacy of anti-fibrotic agents [80–82]. The PCLSs containing both HCC tissues and surrounding normal (or non-tumoral) liver tissues form an ideal model for determining the efficacy and specificity of anti-cancer drugs on a histologically diverse population of cells. PCLSs from various portions of the same tumor are a good model for studying the impact of ITH on treatment responses. Likewise, PCLSs from before and after chemotherapy may provide a useful model for studying spatiotemporal heterogeneity and deducing the therapeutic response.

## Liquid biopsy

Liquid biopsy is emerging as an alternative to tumor tissue biopsy. It allows the circulating tumor cells (CTCs), nucleic acids from the tumors including circulating tumor RNA (ctRNA) and circulating tumor DNA (ctDNA), as well as other tumor biomarkers in the bloodstream and other body fluids to be studied [83–85]. These materials shed from the primary and/or metastatic tumors and represent their heterogeneity. In particular, the presence of a heterogeneous population of CTCs in the blood indicates tumor heterogeneity at the phenotypic and genotypic levels [86, 87]. In fact, the phenotypic characterization of CTCs can offer insights into treatment choices, with some CTC phenotypes linked to drug sensitivity, including pERK/pAkt and PD-L1-positive CTCs [88, 89]. In addition, the level of CTCs and their phenotypic characteristics can enhance our understanding of metastatic biology and mechanisms of drug resistance. In given HCC patients, spatial analysis of CTCs from different vessels can predict metastases [90]. Taken together, liquid biopsy not only provides valuable insights into tumor heterogeneity but also holds promise for predicting metastases through spatial analysis of CTCs.

## Conclusion and prospects

Cellular and molecular heterogeneity contribute significantly to the late diagnosis, drug resistance, and treatment failure in HCC. Previous techniques such as bulk RNA sequencing, whole genome sequencing, and scRNA-seq have been extremely useful in deriving different cell types in HCC. However, more clinically relevant and translational study models that can faithfully mimic the native tumors will advance the understanding of the mechanisms of HCC heterogeneity and its impact on the development of drug resistance and therapy failure. More studies using advanced translational models in large cohorts of patients of varying etiological, ethnic, and genetic backgrounds may facilitate the identification of critical biomarkers for accurate early diagnosis of HCC and lead to efficient treatments.
